# Dissociation of Cross-Sectional Trajectories for Verbal and Visuo-Spatial Working Memory Development in Rubinstein-Taybi Syndrome

**DOI:** 10.1007/s10803-016-2736-2

**Published:** 2016-03-24

**Authors:** Jane Waite, Sarah R. Beck, Mary Heald, Laurie Powis, Chris Oliver

**Affiliations:** School of Psychology, Cerebra Centre for Neurodevelopmental Disorders, University of Birmingham, Edgbaston, Birmingham, B15 2TT UK; Specialist Learning Disability and Forensic Services, Hertfordshire Partnership NHS Foundation Trust, Hemel Hempstead, UK; School of Psychology, University of Birmingham, Birmingham, UK

**Keywords:** Working memory, Short-term memory, Rubinstein–Taybi syndrome, Typically developing children, Dissociation

## Abstract

Working memory (WM) impairments might amplify behavioural difference in genetic syndromes. Murine models of Rubinstein–Taybi syndrome (RTS) evidence memory impairments but there is limited research on memory in RTS. Individuals with RTS and typically developing children completed WM tasks, with participants with RTS completing an IQ assessment and parents/carers completing the Vineland Adaptive Behavior Scales. A cross-sectional trajectory analysis was conducted. There were significant WM span deficits in RTS relative to mental age. Verbal WM span was positively associated with mental age; however, this was not observed for visuo-spatial span. There is a dissociation between WM domains in RTS. Individuals may have difficulties with tasks relying on WM span, above difficulties predicted by overall ability.

A growing body of research identifies impairments of executive functions (EFs) as relevant to explaining behavioural difference in people with genetic disorders (Woodcock et al. 2009). Studies of associations between specific cognitive profiles and behaviour can elucidate possible pathways from genetic disorder to behaviour via atypical brain development and interactions with the environment (Woodcock et al. 2011). One component of EF that warrants further investigation is working memory (WM) (Wang and Bellugi 1994).

WM is served by two slave information processing systems: the visuo-spatial sketchpad processes visual and spatial information and the phonological loop processes verbal information (Baddeley and Hitch 1974). A distinction is often made between simple and complex WM tasks. Garon et al. (2008) define simple WM tasks as tasks requiring a person to hold information in mind in either of these systems (synonymous with short-term memory; STM), while complex WM tasks require information to be to manipulated and updated in WM. Compromised simple or complex WM impact on the ability to act purposefully, learn effectively and accomplish goals (Baddeley 1986).

There is evidence that these core information processing systems (phonological loop and visuo-spatial sketchpad) can be differentially impaired; lending support for the separation of these systems in the classic model of WM (Wang and Bellugi 1994). For example, individuals with localised brain injury have shown greater impairment to one system (Hanley et al. 1991), and interference from competing cognitive tasks can impact these systems differentially (Logie et al. 1990). In addition, dissociations have been evidenced in the visuo-spatial sketchpad between the processing of visual and spatial information (Vicari et al. 2004).

Wang and Bellugi (1994) argued that one approach to studying these dissociations is to explore WM profiles in rare genetic syndromes. WM has been studied in rare genetic syndromes and dissociations are reported. Jarrold et al. (1999) found that individuals with William syndrome performed poorly on simple visuo-spatial WM tasks relative to mental age (MA). However, relative strengths were evident for simple phonological WM tasks. The opposite pattern was found for Down syndrome. Other syndromes might further inform this potential dissociation. A relatively neglected syndrome in which WM impairments are implicated from murine models but not yet explored in humans is Rubinstein–Taybi syndrome (RTS).

RTS is a multiple congenital anomaly syndrome estimated to occur in 1:100,000 to 1:125,000 live births and most often associated with chromosome 16p.13.3; however, genetic diagnosis is only possible in around 55 % of individuals. The majority of diagnoses are based on the physical phenotype that includes short stature, downward slanting perebral fissures, short “beaked” nose and broad thumbs and toes (Hennekam 2006). Behavioural characteristics include insistence on sameness, adherence to routine and repetitive questions (Waite et al. 2015), with tentative evidence of heightened social interest in RTS in comparison to individuals matched on developmental level (Galéra et al. 2009). Intellectual disability (ID) ranges from mild to severe, with expressive language delayed (Clarke and Langton 1992). Few cognitive and behavioural differences have been identified between those with and without a genetically confirmed diagnosis (Bartsch et al. 1999). Murine models have led to the proposal that ID may be underpinned or exacerbated by impaired learning due to long-term memory (LTM) deficits (Oike et al. 1999; Weeber and Sweatt 2002; Wood et al. 2005); however, no published studies have systematically investigated memory.

While it has been hypothesised that impaired LTM underlies compromises learning in RTS, LTM is a single component of a broader memory system. Models of memory highlight that LTM interacts with WM. The successful interaction of WM and LTM processes contributes to knowledge acquisition and problem solving (Logie 1996). Additionally, WM and set-shifting have been linked to repetitive behaviours, and an inability to recall words on a STM task has been linked to perseverative speech in dementia (Woodcock et al. 2009; Turner 1999; Cullen et al. 2005). In RTS, elevated levels of repetitive questioning have been noted (Waite et al. 2015). One possibility is that WM deficits are associated with repetitive questions. In this study we focus on WM as the first step toward developing a model of compromised memory underpinning impaired learning and behavioural characteristics in RTS.

Studying WM development in RTS requires an appropriate comparison group. In TD children, WM has been associated with the development of various abilities including: vocabulary acquisition, reading comprehension, mathematics, decision making and theory of mind (Bull et al. 2008; Engle et al. 1999; Cain et al. 2004; Carlson et al. 2002; Baddeley 1986). Understanding the WM profile of RTS relative to TD children may lead to more specific hypothesises concerning the relationship between memory and other cognitive abilities in this syndrome. This approach also enables consideration of whether individuals with RTS have WM impairments aligned with global MA or whether they have a profile of strengths and weaknesses relative to MA. The principal aim of this study was to explore the cross-sectional developmental trajectories of working memory domains in RTS in comparison to TD children.

## Methods

### Participants

#### RTS

Thirty-two participants with RTS were recruited (16 males; mean chronological age: 221 months; chronological age range 46–533 months; SD: 121.03). Of these, twenty-seven were recruited from an existing database held by the Cerebra Centre for Neurodevelopmental Disorders and five via the RTS UK Support Group. Participants were included if they were mobile and had a confirmed clinical diagnosis.

Eleven participants were excluded from analysis of the WM tasks because they could not comprehend the task instructions due to young age and/or severity of ID, or because MA fell outside the range of the TD comparison group. The mean chronological age of the remaining 21 participants was 232 months (9 males; age range 81–453 months; SD: 104.66). Of these participants, one did not complete the Verbal Animal Span task due to poor engagement.

#### TD Children

The TD comparison group comprised eighty-nine children (mean chronological age: 62 months; 40 males; range 38–89 months; SD: 15.10) tested in schools in the West Midlands, UK. Participants were included if they were not identified by their class teacher as having a developmental disability. To ensure a spread of ages, where possible, eight TD children were tested in each 6 month age band between 38 and 89 months. TD data for the Scrambled Boxes tasks were not collected beyond 78 months as the task was not deemed developmentally appropriate.

### Measures

As moderate to severe ID is characteristic of RTS, delayed EF development relative to chronological age would be expected. Therefore, assessments were administered to explore whether EFs were delayed/deviant relative to global cognitive development (MA). Individuals with RTS completed assessments of cognitive ability to ascertain MA and three WM tasks.

#### Measures of General Cognitive Functioning

Participants with RTS completed the Mullen Scales of Early Learning (MSEL: Mullen 1995), suitable for individuals from birth to 68 months. Participants at ceiling on the MSEL completed the Wechsler Abbreviated Scales of Intelligence—Second Edition (WASI-II: Weschler 1999), suitable for individuals from 72 months–89 years. Standardised scores could not be derived for many participants because, due to degree of ID, individuals completed the MSEL despite being older than 68 months. MA equivalent scores were calculated for the MSEL by calculating the average of subscale scores from the receptive and expressive language, visual reception and fine motor domains (Richler et al. 2010). The gross motor domain was omitted as the highest obtainable MA on this scale was lower than the other scales. Similarly, MA was calculated for the Wechsler Abbreviated Scales of Intelligence (WASI-II), by averaging the MAs across sub-domains.

#### Adaptive Behaviour Assessment

The Vineland Adaptive Behavior Scales—Second Edition (VABS-II; Sparrow et al. 2005) was included as an alternative measure of MA. This is a parent report measure of adaptive functioning. There are no guidelines for computing global MA for the VABS. In the same manner as for the psychometric assessments, global MA was calculated by taking an average across the nine primary domains.

#### WM Test Selection and Administration

Tasks were selected from the developmental literature and adapted to reduce receptive language demands. Two simple WM tasks, the Verbal Animal Span and Corsi Blocks, were included because pilot work indicated that individuals with RTS had difficulty comprehending the rules for complex WM tasks. One complex WM task, the Scrambled Boxes, was included as it is suitable for very young children (Carlson 2005). The WM tasks were administered as part of a battery of EF tests constructed for a wider research project and were administered in a fixed order. Deviations from this order occurred for six participants who had difficultly engaging with the verbal task first (order: Corsi Blocks, Scrambled Boxes, Verbal Span). No significant differences were found on task scores between these participants and participants who completed the verbal span task first (*ps* > .05).

#### Corsi Blocks (Pickering et al. 1998)

Participants were presented with a 20 × 25 cm white board with ten 3.4 × 3.4 cm blue blocks mounted irregularly. On each trial the researcher touched a sequence of blocks starting with sequences of two. Participants responded by touching the same sequence of blocks. After two practise trials of two block sequences feedback was given. Every three experimental trials the number of blocks in a sequence increased by one. The task was terminated after three consecutive incorrect trials. An adapted version of a one point per pair coding scheme was adopted (Fudala et al. 1974). For example, if the sequence was block 3, block 6, block 7, block 2 and the response given is block 3, block 6, block 7, block 2 then the paired item score was 3 (i.e. 3–6, 6–7 and 7–2). If the response was block 3, block 6, block 3, block 7 the paired score would be 1. Only participants able to point to at least one block correctly on each practise trial and who attempted to locate two blocks in the correct order (demonstrating rule understanding) completed experimental trials.

#### Verbal Animal Span (Adapted from Digit Span, Bull et al. 2004)

This task followed the same protocol and coding as the Corsi Blocks task except participants verbally repeated strings of animal names (all one syllable) after the experimenter said them. This task was adapted from the traditional digit span for individuals less familiar with numbers.

#### Scrambled Boxes Task (Adapted from Diamond 1990)

Three versions of this task were included: Three, Six and Nine Scrambled Boxes. The test equipment was eighteen round wooden boxes (diameter = 7 cm) each decorated with a different shape, nine foam stars, a cardboard treasure chest, a 29.7 × 42 cm cardboard screen and two cardboard baseboards that indicated where the boxes should be positioned in each task. Boxes were positioned 5 cm apart for the Three Scrambled Boxes and 8 cm apart for the Six and Nine Scrambled Boxes task respectively. In all versions, participants watched the experimenter put a star in each box and close them. Participants were asked to find stars and put them in a treasure chest. Once a box was selected and the star removed, the empty box was returned, the boxes were hidden behind the screen and the positions of the boxes were scrambled by the researcher. The boxes were scrambled for 5 and 10 seconds in the three and six/nine Scrambled Box task respectively. Participants then searched again.

The Six Scrambled Boxes task was administered first. If a participant retrieved all six stars without error the task was repeated using nine boxes and a full score was given for the three box task. If an error was made the task was repeated with three boxes and a score of zero given on the nine box task. Maximum scores for the Three, Six and Nine Scrambled Boxes tasks were four, seven and ten respectively, with one point lost for each incorrect reach. The task was terminated if the participant lost all their points. A composite scaled score was calculated by summing scores from the three tasks.

## Data Analysis

### Validity of MA Equivalent Scores and Association with CA

Mean age equivalent score for the total RTS group (N = 32) on the direct cognitive assessments (MSEL and WASI) was 61.83 months (*SD*: 34.20). Mean age equivalent score on the indirect informant report measure (VABS-II) was 65.89 months (*SD*: 37.16). A Wilcoxon test revealed no significant differences between these scores. The intraclass correlation coefficient between the direct and indirect MA equivalent scores was calculated to measure the level of agreement: .91 (95 % CI Lower = .82, Upper = .96, (df: 30, 30), F = 21.41, *p* < .001). Given the high level of convergence between MA equivalent estimates, only scores from the direct assessments (MSEL and WASI) were used in further analyses.

To aid interpretation of MA cross-sectional trajectories a linear regression was conducted to explore associations between MA (MSEL and WASI) and CA in RTS. A straight line fitted these data (R^2^ = .41, F(1,30) = 20.50, *p* < .001) with an intercept of 4.18 and a gradient of 0.27; 95 % CI 2.31–5.83).

### Analysis of WM Tasks

As development is a dynamic process, traditional group comparisons that match a syndrome group to a control group can obscure important changes in the cross-sectional developmental trajectory of the syndrome group (Karmiloff-Smith 1998; Thomas et al. 2009). For example, if there is a peak in performance at a particular age followed by a decline this may be obscured when a group average is taken. Thomas et al. (2009), Thomas (2010) described how linear cross-sectional trajectory analysis, involving the graphical representation of all data points, can aid understanding cognitive development whilst overcoming the limitations of matching. This methodology was applied to data obtained from the simple WM tasks. Data from the Scrambled Boxes task were not appropriate for linear cross-sectional trajectory analysis so independent t-tests were conducted, with an alpha level of .01 to correct for multiple tests.

Prior to the between groups linear cross-sectional trajectory analysis, regression lines were fitted to the simple WM task data for each group. Between Groups linear cross-sectional trajectory analysis compares the intercepts (onset of the lines) and gradients (slopes of the lines) of two cross-sectional trajectories that are plotted as a function of age to ascertain whether the trajectories differ for the two groups at the earliest age of measurement (equivalent of a main effect of group), and whether age may differentially impact on the two groups. The analysis was conducted as described by Thomas et al. (2009), Thomas (2010) by making an adaption to the Analysis of Covariance function within General Linear Model (ANCOVA). Typically, including two groups with different cross-sectional trajectories in an ANCOVA is a violation of the test’s assumptions because ANCOVA computes one regression function during the analysis; however, by adding an interaction term to this model (group × age) it is possible to compare the slope of the two cross-sectional trajectories. The x-axis was rescaled prior to the analysis so that the intercept of the regression lines would represent scores at the youngest age of measurement. Further details on this method are available at: http://www.psyc.bbk.ac.uk/research/DNL/stats/Thomas_trajectories.

## Results

**Fig. 1:**
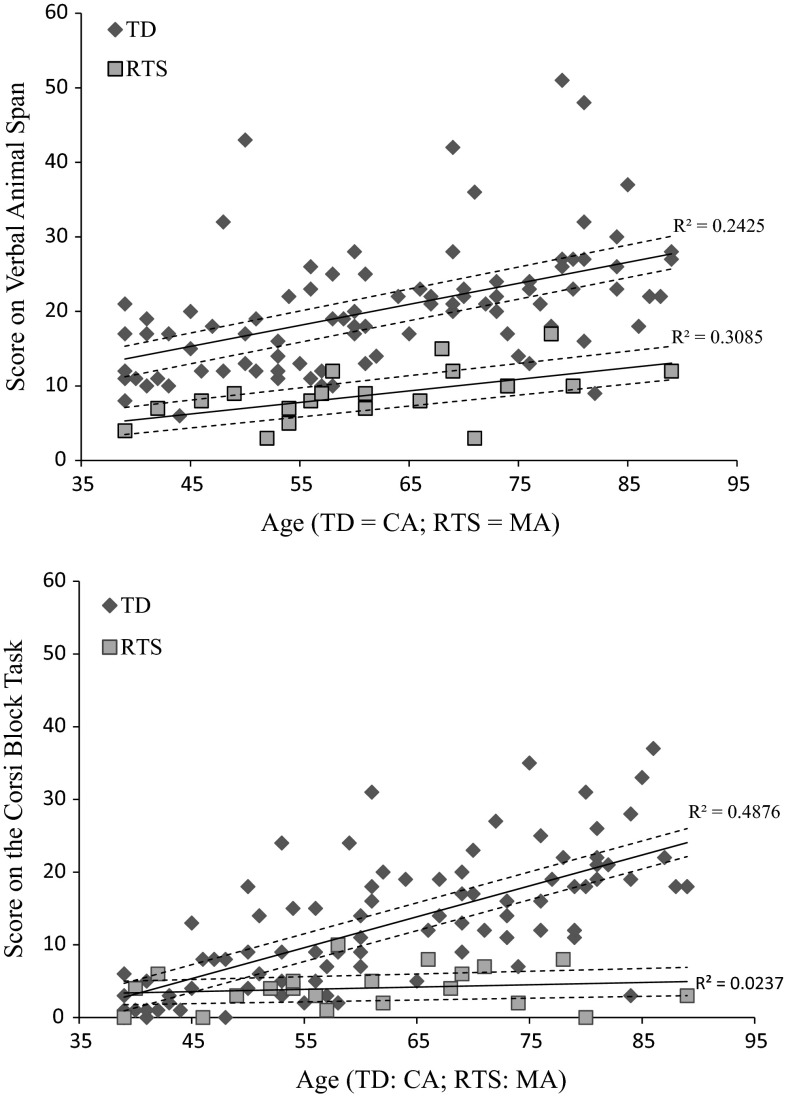
RTS and TD trajectories for scores on the span tasks as a function of MA

The descriptive statistics for the WM tasks are displayed in Table 1 with linear cross-sectional trajectories for the Verbal Animal Span and Corsi Blocks displayed in Fig. 1.

**Table 1:** Descriptive statistics (mean, SD & range) on working memory tasks for RTS and TD group

	RTS (N = 21)		TD (N = 89)	
	Mean (SD)	Range	Mean (SD)	Range
Verbal animal span	8.75 (3.67)	0–17	20.36 (8.56)	6–51
Corsi blocks task	4.05 (2.78)	0–10	12.98 (9.09)	0–37
Scrambled boxes task—Composite Score	8.14 (5.72)	4–21	10.95 (5.50)	2–21

### Verbal Animal Span Cross-Sectional Trajectory

Initial regression analyses indicated that a straight line fitted the RTS data, R^2^ = .31, F(1, 18) = 8.04, *p* = .01, with an intercept of 5.30 and gradient of 0.16, and the TD data, R^2^ = .24, F(1, 87) = 27.85, *p* < .001, with an intercept of 13.61 and gradient of 0.28.

The adapted ANCOVA indicated that a significant proportion of the overall variance was explained by this model, F(3,105) = 26.59, *p* < .001, = .43. There was a 8.31 point score difference between the intercepts of the TD and RTS and TD trajectories, F(1,105) = 5.95, *p* = .016, η^2^ = .05. When the TD and RTS groups were combined, MA significantly predicted score on the Verbal Animal Span, F(1,105) = 11.17, *p* = .001, η^2^ = .10; however, there was no significant age x group interaction, F(1,105) = .94, *p* = .334, η^2^ = .01. Thus, the significant score difference between the groups remained consistent across the age range.

### Corsi Blocks Cross-Sectional Trajectory Analysis

Linear regression analyses revealed that a straight line fitted the TD Corsi Blocks data (R^2^ = .49, F(1, 87) = 82.78, *p* < .001), with an intercept of 2.81 and gradient of .43. A straight line did not fit the RTS data [R^2^ = .02, F(1, 19) = 0.46, *p* = .505] but this was due to the flat cross-sectional trajectory (see Fig. 1). The cross-sectional trajectory had intercept of 3.38 and gradient of .05.

The adapted ANCOVA indicated that a significant proportion of the overall variance was explained by this model, F(3,106) = .17.79, *p* < .001, = .14. The RTS and TD scores were not significantly different at the youngest age of measurement (intercepts) on the cross-sectional trajectory, F(1,106) = .05, *p* = .837, η^2^ < .01. There was significant group × age interaction, F(1,106) = 13.23, *p* < .001, η^2^ = 0.11. The RTS cross-sectional trajectory appears flat, while the TD cross-sectional trajectory has a positive slope with age (see Fig. 1).

The point at which the 95 % confidence intervals no longer overlap (see Fig. 1) indicates that the cross-section trajectories are reliably different at 49 months.

#### Scrambled Boxes Analysis

There were no significant differences between the RTS and TD groups on the Three Scrambled Boxes, t(22.65) = 1.54, *p* = .137, *d* = 0.22, Six Scrambled Boxes, t(25.02) = 1.78, *p* = .087, *d* = 0.50, or Nine Scrambled Boxes, t(38.20) = 1.78, *p* = .140, *d* = 0.33. Using our adjusted alpha level the total score also failed to reach significance, t(107) = 2.31, *p* = .039, *d* = 0.50. In addition, no significant correlations between performance and MA were found.

## Discussion

This study explored the development of WM in RTS relative to MA using cross-sectional trajectory methods Thomas et al. (2009), Thomas (2010). MA was calculated by averaging MA equivalent domain scores from the MSEL and, while this method is likely to only provide a gross estimate of MA, the MAs appeared to have convergent validity with MA estimates from an informant assessment (VABS). Cross-sectional trajectories were then presented for verbal and spatial span tasks. The results indicated that WM span may be compromised in RTS but performance was variable across tasks depending on the aspect of WM measured.

Findings suggest that in RTS verbal and visuo-spatial WM span may be compromised relative to MA. This is illustrated by the Animal Span task and the Corsi Blocks cross-sectional trajectories as performance on these tasks is below that of the TD group. Despite this, there are some differences between cross-sectional trajectories. On the verbal span task, TD children consistently outperform the individuals with RTS. While the RTS group lags behind the TD group, individuals with RTS who had higher MAs performed better than those with lower MAs. The design is cross sectional so change over time cannot be assumed; however, a moderate association between MA and chronological age suggests improvement in verbal span with chronological age in RTS. The Corsi block span shows a different pattern whereby there is initial overlap of the RTS and TD cross-sectional trajectories at the youngest age of measurement (MA) but the RTS trajectory remains flat in contrast to the positive slope for the TD group. In addition, a proportion of individuals with RTS were not able to score on the experimental trials of this task (requiring them to retain two items in memory) despite understanding the rules of the task and memorising at least one block during the practise phase.

Not all of the results suggest WM impairments in RTS because the groups did not differ on the Scrambled Boxes task, a visuo-spatial WM task. During this task participants are required to remember distinct objects that vary on two memorable dimensions (colour and shape) and this may be less demanding than the Corsi block task that requires the tracking of movement. There is evidence that tracking movement has different neurological correlates than remembering shape and colour, so these results may represent a dissociation of the visuo-spatial sketchpad (Vicari et al. 2003; Logie 1996). Alternative interpretations are that interacting with the boxes for a longer time or the immediate reward from receiving stars may form stronger memory representations (Vogel et al. 2006), or that the recognisable shapes on the boxes led to some verbal encoding and aided performance. Finally, a conservative alpha level was used in the scrambled boxes analysis to correct for multiple tests, so it remains possible that group differences could exist on this task. As with all these tasks, further investigation is necessary to extrapolate to the mechanisms underlying performance.

As noted previously, it has been proposed that ID associated with RTS may be linked to mutations in the CREB binding protein and the effects on LTM associated with hippocampal functioning (Oike et al. 1999; Weeber and Sweatt 2002; Wood et al. 2005). A number of studies with knock-out mice have explored the link between these mutations and phenotypic characteristics, and while these mice develop LTM difficulties, STM is not affected (see Josselyn (2005) for a review). It has been be argued that simple WM tasks, such as those included in the current study, can only be defined as STM tasks because of the absence of an updating component (Gathercole and Alloway 2006). Therefore, the poor performance of the RTS group on simple WM tasks does not fit neatly with murine models of RTS. Instead, these results point to a possible double deficit of memory function.

A syndrome comparison group was not included in this study but the RTS memory profile is likely to be phenotypic because WM lags behind overall ability. The memory profile in RTS appears different to other syndrome groups. For example, in Down Syndrome visuo-spatial skills are a relative strength, whereas in William syndrome they are a weakness relative to verbal skills (Jarrold et al. 1999). Individuals with RTS evidence difficulties in both domains.

The results of this study will inform clinicians and teachers working with RTS. External memory aids may be particularly useful for helping individuals remember information sequences and it may be helpful to present information in no more than two–three chunks at a time. The results suggest that older individuals with RTS are likely to have more developed verbal WM spans and further studies could explore the possibility of accelerating development of verbal WM capacity using computerised tasks that train this ability, as has been demonstrated previously (e.g. Klingberg et al. 2005). Finally, it would be interesting to consider WM deficits in RTS in relation to other aspects of the behavioural phenotype such as the high levels of repetitive questioning noted in this group (Waite et al. 2015).

As this is the first study of memory in individuals with RTS, there are inevitably some limitations. Firstly, despite the convergence of MA across assessments, MA can only be taken as an estimate for examining gross dissociations in cross-sectional trajectories at group level. In addition, it is only possible to draw conclusions within the developmental window between 38 and 89 months. Performance of individuals with MAs outside this window may not map onto these cross-sectional trajectories. In addition, participants with RTS would need to be followed up to confirm whether higher performance on verbal span tasks in those with higher MA represents developmental change. Finally, the MSEL and VABS were not completed by the TD comparison group due to constraints of testing in schools. However, the sample of children was large, increasing the likelihood of a MA cross-sectional trajectory accurately reflecting the ability of the TD group.

Order effects may have occurred from fixed order administration. It could be argued that poor performance on the Corsi span task represents general fatigue and disengagement. There was no statistical difference, however, between the small subset of individuals who received the Corsi block span first. Furthermore, all 21 participants went on to complete a broader EF battery as part of a wide scale study without demonstrating a drop off in performance that characterised the Corsi block span; therefore, these results appear robust. A further limitation of the Corsi block span is that it may not discriminate well between the two groups at the youngest ages since all children were at or near the floor of the task. Overall, these results are an encouraging first step towards profiling memory in a syndrome in which memory impairments would be anticipated given murine models (Oike et al. 1999; Wood et al. 2005). In addition, these results lend further support to a dissociation of the phonological loop and visuo-spatial sketchpad. A difference in performance also was found between groups on the Corsi Blocks task but not the Scrambled Boxes task. Whilst these differences may be due to the nature of the tasks used, this provides tentative evidence of a dissociation between spatial processing (e.g. tracking movement) and visual processing in the (e.g. colours and shapes) in the visual spatial sketchpad and concurs with previous research (Logie 1996, Vicari et al. 2003). Exploring the neurological correlates of performance on these WM tasks in individuals with RTS could provide further evidence for the dissociation of these abilities (Vicari et al. 2003).

## References

[CR1] Baddeley A (1986). Working memory.

[CR2] Baddeley A, Gathercole S, Papagno C (1998). The phonological loop as a language learning device. Psychological Review.

[CR3] Baddeley AD, Hitch GJ, Bower GH (1974). Working memory. The psychology of learning and motivation: Advances in research and theory.

[CR4] Bartsch O, Wagner A, Hinkel GK, Krebs P, Stumm M, Schmalenberger B (1999). FISH studies in 45 patients with Rubinstein–Taybi syndrome: Deletions associated with polysplenia, hypoplastic left heart and death in infancy. European Journal of Human Genetics.

[CR5] Bull R, Espy KA, Senn TE (2004). A comparison of performance on the Towers of London and Hanoi in young children. Journal of Child Psychology and Psychiatry.

[CR6] Bull R, Espy KA, Wiebe SA (2008). Short-term memory, working memory, and executive functioning in preschoolers: Longitudinal predictors of mathematical achievement at 7 years. Developmental Neuropsychology.

[CR7] Cain K, Oakhill J, Bryant P (2004). Children’s reading comprehension ability: Concurrent prediction by working memory, verbal ability, and component skills. Journal of Educational Psychology.

[CR8] Carlson SM (2005). Developmentally sensitive measures of executive function in preschool children. Developmental Neuropsychology.

[CR9] Carlson SM, Moses LJ, Breton C (2002). How specific is the relation between executive function and theory of mind? Contributions of inhibitory control and working memory. Infant and Child Development.

[CR10] Clarke, D., & Langton, J. (1992). The Rubinstein–Taybi behavioural phenotype: A postal questionnaire survey. *Paper presented at the 2nd international sumposium of the study of behaioural phenotypes,* Welshpool.

[CR11] Cullen B, Coen RF, Lynch CA, Cunningham CJ, Coakley D, Robertson IH (2005). Repetitive behaviour in Alzheimer’s disease: Description, correlates and functions. International Journal of Geriatric Psychiatry.

[CR12] Diamond A (1990). Developmental time course in human infants and infant monkeys, and the neural bases, of inhibitory control in reaching. Annals of the New York Academy of Sciences.

[CR13] Engle RW, Laughlin JE, Tuholski SW, Conway AR (1999). Working memory, short-term memory, and general fluid intelligence: A latent-variable approach. Journal of Experimental Psychology.

[CR14] Fudala JB, Kunze LV, Ross JD (1974). Auditory pointing test manual.

[CR15] Galéra C, Taupiac E, Fraisse S, Naudion S, Toussaint E, Rooryck-Thambo C (2009). Socio-behavioral characteristics *of children with* Rubinstein–Taybi syndrome. Journal of Autism and Developmental Disorders.

[CR16] Garon N, Bryson SE, Smith IM (2008). Executive function in preschoolers: A review using an integrative framework. Psychological Bulletin.

[CR17] Gathercole SE, Alloway TP (2006). Practitioner review: Short-term and working memory impairments in neurodevelopmental disorders: Diagnosis and remedial support. Journal of Child Psychology and Psychiatry.

[CR18] Hanley JR, Young AW, Pearson NA (1991). Impairment of the visuo-spatial sketch pad. The quarterly Journal of experimental Psychology.

[CR19] Hennekam RC (2006). Rubinstein–Taybi syndrome. European Journal of Human Genetics.

[CR20] Jarrold C, Baddeley AD (2001). Short-term memory in Down syndrome: Applying the working memory model. Down Syndrome Research and Practice.

[CR21] Jarrold C, Baddeley AD, Hewes AK (1999). Genetically dissociated components of working memory evidence from Downs and Williams syndrome. Neuropsychologia.

[CR22] Josselyn SA (2005). What’s right with my mouse model? New insights into the molecular and cellular basis of cognition from mouse models of Rubinstein–Taybi Syndrome. Learning and Memory.

[CR23] Karmiloff-Smith A (1998). Development itself is the key to understanding developmental disorders. Trends in Cognitive Sciences.

[CR24] Klingberg T, Fernell E, Olesen P, Johnson M, Gustafsson P, Dahlström K (2005). Computerized training of working memory in children with ADHD—A randomized, controlled trial. Journal of the American Academy of Child and Adolescent Psychiatry.

[CR25] Logie RH, Richardson JT, Engle RW, Hasher L, Logie RH (1996). The seven stages of working memory. Working memory and human cognition.

[CR26] Logie RH, Zucco GM, Baddeley AD (1990). Interference with visual short-term memory. Acta Psychologica.

[CR27] Mullen EM (1995). Mullen scales of early learning: AGS edition.

[CR28] Oike Y, Hata A, Mamiya T, Kaname T, Noda Y, Suzuki M (1999). Truncated CBP protein leads to classical Rubinstein–Taybi syndrome phenotypes in mice: Implications for a dominant-negative mechanism. Human Molecular Genetics.

[CR29] Pickering S, Gathercole S, Peaker S (1998). Verbal visuospatial short-term memory in children: Evidence for common and distinct mechanisms. Memory and Cognition.

[CR30] Richler J, Bishop SL, Kleinke JR, Lord C (2010). Restricted and repetitive behaviors in young children with Autism Spectrum Disorders. Development and Psychopathology.

[CR31] Sparrow S, Cicchetti D, Balla D (2005). Vineland adaptive behavior scales.

[CR32] Thomas MS, Annaz D, Ansari D, Scerif G, Jarrold C, Karmiloff-Smith A (2009). Using developmental trajectories to understand developmental disorders. Journal of Speech, Language and Hearing Research.

[CR33] Thomas, M. (2010, April). *Worksheet on using SPSS to analyze and compare*-*cross sectional developmental trajectories.* Retrieved May 15, 2010, from http://www.psyc.bbk.ac.uk/research/DNL/stats/Thomas_trajectories.html.

[CR34] Turner MA (1999). Annotation: Repetitive behavior in autism: A review of psychological research. Journal of Child Psychology and Psychiatry.

[CR35] Vicari S, Bellucci S, Carlesimo GA (2003). Visual and spatial working memory dissociation: Evidence from Williams syndrome. Developmental Medicine and Child Neurology.

[CR200] Vicari S, Bates E, Caselli MC, Pasqualetti P, Gagliardi C, Tonucci F, Volterra V (2004). Neuropsychological profile of Italians with Williams syndrome: An example of a dissociation between language and cognition?. Journal of the International Neuropsychological Society.

[CR36] Vogel EK, Woodman GF, Luck SJ (2006). The time course of consolidation in visual working memory. Journal of Experimental Psychology: Human Perception and Performance.

[CR37] Waite J, Moss J, Beck SR, Richards C, Nelson L, Arron K, Burbidge C, Berg K, Oliver C (2015). Repetitive Behavior in Rubinstein–Taybi Syndrome: Parallels with autism spectrum phenomenology. Journal of Autism and Developmental Disorders.

[CR38] Wang PP, Bellugi U (1994). Evidence from two genetic syndromes for a dissociation between verbal and visual-spatial short-term memory. Journal of Clinical and Experimental Neuropsychology.

[CR100] Weeber EJ, Sweatt JD (2002). Molecular neurobiology of human cognition. Neuron.

[CR39] Weschler D (1999). Wechsler abbreviated scale of intelligence.

[CR40] Wood MA, Kaplan MP, Park A, Blanchard EJ, Oliveira AN, Lombardi TL (2005). Transgenic mice expressing a truncated form of CREB-binding protein (CBP) exhibit deficits in hippocampal synaptic plasticity and memory storage. Learning and Memory.

[CR41] Woodcock KA, Oliver C, Humphreys GW (2009). Hypothesis: A specific pathway can be identified between genetic characteristics and behaviour profiles in Prader–Willi syndrome via cognitive, environmental and physiological mechanisms. Journal of Intellectual Disability Research.

[CR42] Woodcock KA, Oliver C, Humphreys GW (2011). The relationship between specific cognitive impairment and behaviour in Prader–Willi syndrome. Journal of Intellectual Disability Research.

